# New challenges in horticultural IPM: artificial intelligence can enhance the use of biocontrol agents

**DOI:** 10.12688/openreseurope.23884.1

**Published:** 2026-06-15

**Authors:** Andrea Lovazzano

**Affiliations:** 1 Dipartimento di Scienze Agrarie Alimentari e Agro-ambientali, Universita degli Studi di Pisa, Pisa, Tuscany, 56124, Italy

**Keywords:** natural enemies; sustainability; agroecosystem; plant health; robotics; DSS; machine learning; computer vision

## Abstract

Modern horticulture faces the dual imperatives of increasing global food production while mitigating the severe environmental and health impacts of conventional chemical pesticides. Biological control agents (BCAs), which utilize beneficial microorganisms, macroorganisms, semiochemical and botanicals to suppress pathogens, represent a cornerstone of sustainable agriculture but have been historically constrained by inconsistent field performance, high specificity, and slow action. Concurrently, Artificial Intelligence (AI) is emerging as a transformative force in agriculture, offering a powerful suite of tools for data analysis, prediction, and automation. The synergy of BCAs and AI creates advanced strategies where AI directly addresses the inherent limitations of biological control. It can foster the deployment of AI-driven predictive models for proactive pest outbreak forecasting, enabling timely and effective BCA application. Furthermore, it details the role of precision robotics and drones, guided by computer vision, in the targeted delivery of these agents. These components are synthesized into the concept of Integrated Pest Management (IPM) 5.0, where intelligent decision support systems orchestrate a holistic, data-driven approach to plant health. However, the realization of this vision is contingent on overcoming significant economic, regulatory, and adoption hurdles. High initial costs, complex and divergent regulatory countries in the EU and US, and socio-technical barriers to farmer adoption present formidable challenges. A focus on developing robust, low-cost technologies, enhancing BCA formulation and stability, creating interoperable data frameworks, and addressing the socio-economic factors is necessary to translate technological potential into widespread, sustainable practice.

## Plain Language summary

Modern agriculture must grow more food while reducing harmful chemical pesticides. To achieve this, farmers use Integrated Pest Management (IPM), a strategy that prioritizes environmentally friendly methods. A cornerstone of IPM is biological control, which uses natural enemies to fight crop pests. However, biological control is highly complex and its success can be inconsistent. Furthermore, modern challenges like climate change, pesticide resistance, and invasive species make pest management increasingly difficult.

Artificial Intelligence (AI) can offer powerful solutions to these problems. AI technologies, including computer vision and machine learning, can automatically monitor crops to identify both pests and beneficial insects in real-time. By analyzing weather and farm data, AI can predict pest outbreaks before they happen. This allows farmers to release biological control agents at the exact right time. Additionally, robots and drones guided by AI can deliver these natural enemies precisely where they are needed, reducing waste and labor.

This integration creates a “digital nervous system” for farms, making biological control more reliable and significantly reducing the need for chemical pesticides. While high costs, complex regulations, and the need for diverse training data remain barriers, AI has the transformative potential to make horticultural pest management smarter, more autonomous, and highly sustainable.

## 1. Introduction

### 1.1 The enduring paradigm of integrated pest management (IPM) in horticulture

Horticultural production, a cornerstone of global food security and economic stability, faces a persistent threat from a diverse array of pests, including insects, pathogens, and weeds. Historically, the primary response to these threats has been the intensive application of synthetic chemical pesticides. While offering immediate and often effective control, this overreliance has precipitated a cascade of adverse consequences, including the development of pesticide-resistant pest populations, contamination of soil and water resources, harm to non-target organisms such as pollinators and natural enemies, and risks to human health. These detrimental effects have catalyzed a paradigm shift in crop protection towards more sustainable and ecologically sound strategies.

At the forefront of this shift is Integrated Pest Management (IPM), a holistic, ecosystem-based framework that has become the globally accepted standard for sustainable pest control. IPM is not a singular method but rather a comprehensive, decision-making process that strategically integrates multiple control tactics to manage pest populations below economically damaging levels while minimizing risks to human health and the environment ( Kennedy et al., 2008). The fundamental goal of IPM is the long-term management and prevention of pest problems, rather than the short-term, often unsustainable, goal of pest eradication ( Kogan, 1998).

The implementation of an IPM program is typically structured around a four-tiered approach that guides growers from observation to intervention in a logical, evidence-based sequence. The first step is setting action thresholds, which defines the point at which pest populations or environmental conditions warrant control action. This critical step moves away from arbitrary, calendar-based spraying schedules and ensures that interventions are only made when there is a quantifiable economic or aesthetic threat. The second tier, monitoring and identification, involves regular and systematic scouting of crops to accurately identify pest species and their population levels. Correct identification is paramount, as it prevents the misapplication of control measures and allows for the conservation of innocuous or beneficial organisms that naturally inhabit the agroecosystem. The third tier, prevention, is the first line of defense in any IPM program. It encompasses a range of proactive strategies designed to make the crop environment less conducive to pests. These include cultural methods such as crop rotation, intercropping, sanitation, and the selection of pest-resistant cultivars. These practices are often highly effective and present little to no risk to the environment. The final tier, control, is implemented only when monitoring indicates that pest populations have surpassed the action threshold and preventive measures are no longer sufficient. IPM prioritizes the use of the most effective, yet least risky, control options first. This hierarchy includes physical and mechanical controls (e.g., traps, barriers), biological controls, and highly targeted, selective chemical interventions (e.g., pheromones; Walker, 1988). Broad-spectrum synthetic pesticides are reserved as a last resort, to be used judiciously and only when all other options have been exhausted.

The adoption of IPM has yielded significant environmental, economic, and social benefits. Environmentally, it fosters biodiversity conservation, protects vital ecosystem services like pollination, and reduces the chemical load on the environment, with studies indicating that IPM can decrease pesticide applications by 30–50%. Economically, while it may require an initial investment in knowledge and monitoring, IPM often leads to long-term profitability through reduced input costs, minimized crop losses, and improved market access for produce with lower pesticide residues ( Fantle-Lepczyk et al., 2022). Socially, IPM enhances food safety and quality for consumers and reduces the occupational health risks for agricultural workers who are exposed to pesticides. This multi-faceted approach is central to the sustainable intensification of agriculture, seeking to optimize crop production while preserving the ecological integrity of farming systems for future generations.

### 1.2 Biological control: the cornerstone of sustainable IPM

 Within the IPM framework, biological control (biocontrol) stands out as a cornerstone strategy, embodying the ecological principles that underpin sustainable pest management. Biological control is the deliberate use of living organisms, known as natural enemies or biocontrol agents (BCAs), to suppress the populations of pest organisms ( Chauhan et al., 2018; Lacey et al., 2008, Lacey et al., 2015; Hajek, 2004). This approach leverages natural predator-prey, host-parasitoid, and pathogen-host relationships to maintain pests at levels below the economic injury threshold, thereby reducing the reliance on synthetic chemical interventions and mitigating their associated negative impacts ( Hassell and Waage, 1984; Mills and Getz ,1996) The ecological importance of BCAs is profound; they contribute to the maintenance of ecological balance, conserve biodiversity by targeting specific pests with minimal disruption to non-target species, and enhance the overall resilience of the agroecosystem. BCAs are broadly categorized based on their nature and mode of action, each presenting unique opportunities for integration into horticultural IPM programs. These categories are summarized in  Table 1.

**Table 1.** Key Biocontrol Agent (BCA) categories and their mechanisms.

BCA category	Sub-type/example	Primary mechanism of action	Typical application in horticulture
**Macrobials**	Predators (e.g., *Phytoseiulus persimilis*, ladybugs)	Predation: actively hunt and consume pest organisms.	Augmentative release of live organisms.
	Parasitoids (e.g., *Encarsia formosa*, *Trichogramma* spp.)	Parasitism: lay eggs in or on a host pest, with the developing larvae consuming and killing the host.	Augmentative release of live organisms, often as parasitized host eggs/pupae on cards.
**Microbials**	Entomopathogenic Nematodes (e.g., *Steinernema feltiae*)	Infection: actively seek out insect hosts, enter their body, and release symbiotic bacteria that kill the host.	Drench or spray application of a solution containing nematodes.
	Bacteria (e.g., *Bacillus thuringiensis*, Bt)	Toxin production: produce crystalline proteins that are toxic to specific insect orders upon ingestion.	Spray application of spore/crystal mixture.
	Fungi (e.g., *Beauveria bassiana*, *Trichoderma* spp.)	Infection and competition: pores germinate on the insect cuticle, penetrate the body, and proliferate, killing the host. Some species also act as antagonists to plant pathogens through mycoparasitism and competition.	Spray application of fungal spores.
	Viruses (e.g., Nuclear Polyhedrosis Viruses, NPVs)	Infection: must be ingested by the pest; virus replicates within the host cells, leading to liquefaction and death.	Spray application of viral occlusion bodies.
**Semiochemicals**	Pheromones (e.g., sex pheromones)	Mating disruption and attraction: saturate an area with synthetic pheromones to confuse males and prevent them from locating females, or lure pests into traps for monitoring or mass trapping.	Deployment in passive dispensers, aerosol “puffers,” or specialized traps.
**Natural substances (“Botanicals”)**	Botanical extracts (e.g., Azadirachtin from Neem oil, Sweet orange oil, etc)	Repellency, antifeedant, insecticidal: disrupt insect feeding, growth, and reproduction, or act as direct toxins.	Spray application of extracted and formulated plant oils or compounds.

The mechanisms through which these agents operate are diverse and sophisticated. Macrobials, such as predatory mites and parasitic wasps, are typically released augmentatively into a crop to bolster existing natural enemy populations ( Schmitz and Barton, 2014). Microbials, like *Bacillus thuringiensis* (Bt) and entomopathogenic fungi, are often formulated and applied in a manner similar to chemical pesticides but act through biological processes of infection or intoxication ( Zhang et al., 2023; Brown et al., 2004; Irsad et al., 2023; Díaz-Rodríguez et al., 2025). Fungal and bacterial antagonists also employ strategies like antibiosis (producing inhibitory compounds), competition for nutrients and space, and inducing systemic resistance (ISR) in the host plant, which primes the plant’s own defense mechanisms against future attacks ( Mani et al., 2022). Semiochemicals, particularly sex pheromones, offer a highly specific method of behavioral manipulation, disrupting pest mating cycles with no direct impact on other organisms. Despite the clear advantages of biological control, its widespread adoption has been hampered by a significant practical challenge: complexity. The successful application of biocontrol is considerably more knowledge-intensive than conventional chemical control. Chemical control often follows a straightforward “spray-and-kill” logic, with immediate and visible results that provide a sense of security to the grower. In contrast, biological control involves the management of a dynamic ecological interaction. Its success is contingent upon a multitude of interacting variables, including temperature, humidity, crop architecture, pest density, the timing of BCA releases, and the intricate population dynamics between the pest and its natural enemy. This complexity creates a substantial knowledge barrier for growers, who may lack the specialized training, time, or resources required for the intensive monitoring and ecological analysis necessary to make biocontrol work effectively. This knowledge gap is a primary reason why, despite its profound benefits, biological control has not yet fully supplanted chemical-centric approaches. A real challenge, however, highlights a critical opportunity where modern technology, specifically artificial intelligence, can intervene ( Ray, 1994). AI systems are uniquely capable of processing complex, multi-variable data sets and translating them into simplified, actionable recommendations, thereby lowering the knowledge barrier, and making sophisticated, ecologically-based pest management strategies more accessible to a broader range of horticultural producers ( OECD AI, 2019).

### 1.3 Emerging challenges in the 21 ^st^ century: climate change, resistance, and invasive species

The foundational principles of IPM, while robust, are being tested by a new generation of interconnected and accelerating challenges that define 21 ^st^-century agriculture. These pressures are reshaping pest dynamics, undermining the efficacy of established control tactics, and demanding a more adaptive and intelligent approach to crop protection. The three most formidable of these challenges are climate change, the evolution of pesticide resistance, and the proliferation of invasive species ( Challinor et al., 2014; Croft and Van de Baan, 1988).

Climate change is exerting a profound and multifaceted influence on horticultural agroecosystems ( Bentivoglio et al., 2022). Rising global temperatures, altered precipitation patterns, and an increased frequency of extreme weather events are directly impacting the physiology, behavior, and distribution of both pests and their natural enemies ( Lesk et al., 2016). Warmer winters can lead to higher overwintering survival rates for many pest species, while longer growing seasons may allow for additional pest generations per year, leading to larger and more persistent populations ( Bale and Hayward (2010). Temperature shifts are also causing geographic range expansion, with tropical and subtropical pests migrating into previously temperate zones where crops may lack innate resistance and established natural enemies are absent. Furthermore, climate-induced stress on host plants can make them more susceptible to pest attacks. Crucially, climate change can create asynchrony between pests and their biocontrol agents ( Parmesan and Yohe, 2003); if a pest emerges earlier in the season due to warmer temperatures but its key parasitoid does not, the window for effective biological control can be lost, leading to uncontrolled pest outbreaks. This increased unpredictability makes traditional, experience-based IPM calendars obsolete and necessitates a more dynamic, data-responsive management system.

The second major challenge is the relentless evolution of pesticide resistance. Decades of reliance on a limited number of chemical modes of action have imposed immense selection pressure on pest populations, leading to the widespread development of resistance. This evolutionary response renders previously effective chemical controls partially or completely useless, forcing growers to increase application rates or switch to more toxic, and often more expensive, alternatives (  Hallmann et al., 2014). Resistance has been documented in numerous key horticultural pests and is a primary driver for the failure of conventional pest management programs. This crisis not only increases production costs but also exacerbates the environmental and health risks associated with pesticide use, creating a pressing need for effective non-chemical control strategies like biological control ( Jacobsen, 1997).

Thirdly, the interconnectedness of the global economy, coupled with climate change, has accelerated the introduction and establishment of invasive species ( Mosavi et al., 2018). Non-native pests, introduced to new environments without their co-evolved natural enemies, can experience explosive population growth, causing devastating economic and ecological damage ( Musolin, 2007). Invasive species disrupt established agroecosystems, outcompete native species, and can introduce new plant diseases ( Médiène et al., 2011; Campbell and Madden, 1990). They represent a significant threat to biosecurity and often require costly, large-scale eradication or management programs. Managing these new threats requires rapid detection, accurate identification, and the development of novel control strategies, often involving classical biological control—the introduction of natural enemies from the pest’s native range ( Orr, 2009).

These three challenges do not operate in isolation; they interact synergistically, creating a compounding effect that severely strains horticultural IPM programs. For instance, climate change facilitates the establishment of invasive species in new territories ( Robinet and Roques, 2010). The increased pest pressure resulting from both climate change and new invasions often tempts growers to revert to more intensive pesticide use, which in turn accelerates the development of pesticide resistance in both native and invasive pest populations ( Bale and Hayward, 2010). This creates a vicious cycle: new or more resilient pests lead to more spraying, which leads to greater resistance, further limiting the available control options and increasing the reliance on a shrinking arsenal of effective chemicals. This complex, dynamic, and increasingly unpredictable pest management landscape is rapidly outpacing the capacity of static, manual, and purely reactive IPM strategies ( Bianchi et al., 2006). The successful navigation of these intertwined challenges demands a paradigm shift towards management systems that are more adaptive, data-driven, predictive, and intelligent, a domain where artificial intelligence is poised to make a transformative impact.

### 1.4 The Artificial Intelligence revolution: a new frontier for IPM

In response to the escalating complexities of modern agriculture, a technological revolution is underway, driven by advancements in Artificial Intelligence (AI; Wu et al., 2025). AI, encompassing a suite of technologies including machine learning (ML), deep learning (DL), computer vision, and robotics, is rapidly moving from a theoretical concept to a practical and transformative force in the agricultural sector ( Jindal et al., 2021; Jobin et al., 2019). The market for AI in agriculture is projected to grow exponentially, from $1.7 billion in 2023 to $4.7 billion by 2028, reflecting its immense potential to enhance productivity, sustainability, and profitability.

AI’s core capability lies in its ability to process and analyze vast and complex datasets far beyond human capacity, identifying patterns, making predictions, and automating decisions and actions. In agriculture, this translates into a range of powerful applications ( Omotayo et al., 2025). Precision farming systems leverage AI to analyze data from satellites, drones, and in-field sensors to optimize the application of water, fertilizers, and pesticides on a site-specific basis, maximizing efficiency and minimizing waste ( Chakrabarty et al., 2026). Autonomous robots and drones, guided by AI, can perform tasks such as planting, weeding, and harvesting with high precision, addressing labor shortages, and reducing operational costs ( Cubero et al., 2020; Slaughter et al., 2008). Predictive analytics, powered by machine learning, can forecast crop yields, predict disease outbreaks, and model the impacts of climate change, enabling farmers to move from reactive problem-solving to proactive, strategic management ( Wu et al., 2014; McCown, 2002).

It is within this context that AI emerges as a new and powerful frontier for Integrated Pest Management. The challenges of climate change, pesticide resistance, and invasive species have pushed traditional IPM to its limits, highlighting the need for systems that can handle greater complexity and uncertainty ( Amiri et al., 2024; Sharma, 2014). AI provides the technological toolkit to meet this need. It has the potential to supercharge every tier of the IPM framework by enhancing monitoring, improving prediction, and automating targeted interventions.

The central thesis of this review is that AI does not seek to replace the foundational principles of IPM. Instead, it provides the means to implement them with a level of precision, timeliness, and ecological awareness that was previously unattainable. More specifically, AI is uniquely positioned to overcome the primary barriers limiting the widespread adoption of biological control. By automating the complex tasks of monitoring both pests and beneficials, predicting their dynamic interactions, and optimizing the deployment of biocontrol agents, AI can make these sustainable strategies more reliable, cost-effective, and accessible. This integration promises to shift the focus of IPM away from a reliance on last-resort chemical applications and firmly towards a proactive, preventative system centered on the intelligent use of biological control.

## 2. Analysis of the recent literature

The integration of artificial intelligence into horticultural IPM is catalyzing a fundamental transformation, moving pest management from a practice based on manual observation and reactive treatments to a system driven by data, prediction, and precision. AI technologies are enhancing every stage of the biological control process, from the initial detection of pests and their natural enemies ( Beers et al., 2016) to the optimized, automated deployment of biocontrol agents. This section details the state-of-the-art applications of AI across the key functional areas of surveillance, prediction, intervention, and integrated decision support. A summary of these applications, mapped to the core stages of IPM, is presented in Table 2.

**Table 2.** A brief summary of AI technologies and their application in enhancing biocontrol within the IPM.

IPM Stage	AI Technology	Specific application to Biocontrol
**Monitoring and Identification**	Computer vision (CNNs, YOLO); acoustic and hyperspectral sensing	Real-time, automated identification of pests and beneficial organisms (predators, parasitoids, and their life stages); pre-symptomatic detection of pest-induced plant stress.
**Prediction and analysis**	Machine learning (RNNs, random forests); System dynamics modeling	Forecasting pest outbreaks based on environmental and population data to optimize the timing of BCA releases; Modeling complex predator-prey dynamics to determine optimal release ratios and predict control outcomes.
**Control and intervention**	Robotics (UAVs, UGVs); Precision spraying; Optimization algorithms	Automated, uniform, and rapid release of macrobial BCAs (e.g., predatory mites, parasitoids) over large or difficult-to-access areas; Targeted, plant-specific application of microbial BCAs (e.g., entomopathogenic fungi) to minimize waste and off-target effects; Calculating optimal release rates, timing, and spatial distribution for maximum efficacy and cost-efficiency.

### 2.1 AI-powered pest and natural enemy surveillance

Effective IPM begins with accurate and timely monitoring. Traditionally, this has been a labor-intensive, time-consuming, and often error-prone process, relying on human scouts to manually inspect crops and traps. AI-powered surveillance technologies are automating and enhancing this foundational step, providing growers with unprecedented insights into the dynamic ecology of their fields. This technological shift is enabling a more profound change: moving from simple “pest scouting” to comprehensive “ecosystem monitoring.”

Historically, the primary question in pest monitoring has been, “Is the pest present, and has its population overcome the economic threshold?” This is a fundamentally pest-centric and reactive query. The effective use of biological control, however, requires a more sophisticated and ecological perspective ( Chauhan et al., 2018; Preti et al., 2021). The relevant question becomes, “What is the dynamic balance between pest and natural enemy populations, and how is this ratio likely to evolve under the current and forecasted environmental conditions?” Answering this requires data on both sides of the predator-prey equation. Early AI applications mirrored the traditional approach, focusing on the detection of a single pest species. However, the state-of-the-art has advanced significantly. Modern AI systems are now capable of simultaneously identifying multiple pest species, their various life stages, and, crucially, the beneficial insects that regulate them. This capability fundamentally transforms the nature of monitoring. It allows for the implementation of more ecologically relevant action thresholds, such as predator-to-prey ratios, rather than simple pest counts. This enables a true ecosystem-based approach to pest management, where intervention decisions are based on the health and balance of the local agroecosystem, a core tenet of IPM that has historically been difficult to implement at scale ( Scott-Morton, 1971).

*2.1.1 Computer vision for real-time identification of pests and biocontrol agents*

Computer vision, particularly deep learning, has emerged as the leading technology for automated species identification in agriculture ( Yu & Zheng, 2024; Li et al., 2025;  Shoaib et al., 2025). By training algorithms on large datasets of images, these systems can learn to recognize and differentiate between various organisms with remarkable accuracy ( Russakovsky et al., 2015). Convolutional Neural Networks (CNNs) are a class of deep learning models that have proven exceptionally effective for image-based classification tasks ( Upadhyay et al., 2025). Researchers have developed CNN-based systems that can identify plant diseases and pest damage from leaf images with accuracies often exceeding 95%, surpassing the capabilities of human visual assessment in some cases. These models can be deployed via mobile applications, allowing growers to get instant diagnoses in the field by simply taking a picture of an affected plant. For real-time detection and counting, object detection models such as the “You Only Look Once” (YOLO) family of algorithms are widely used ( Amrani et al., 2023). YOLO and its advanced iterations (e.g., YOLOv4, YOLOv8, YOLOv10) can process images or video streams rapidly, drawing bounding boxes around detected objects and classifying them simultaneously. This technology is ideal for automated monitoring using sticky traps, where a camera can periodically capture an image of the trap, and the YOLO model can identify and count the captured insects. Similarly, some research project are based on the adoption of traps for insects without use of sticky panels. For instance, Insect Detect DIY (do-it-yourself ) camera trap for non-invasive automated monitoring of flower-visiting insects, which is based on low-cost off-the-shelf hardware components combined with open-source software ( Sittinger et al., 2024).

The most significant advancement in this area is the expansion of these systems to identify beneficial organisms alongside pests. This is critical for biocontrol-based IPM, as the presence and abundance of natural enemies directly influence the need for intervention. This dual-monitoring capability provides a much more complete ecological picture, enabling more nuanced and sustainable management decisions ( Tyagi et al., 2024).

Similar systems are being developed for high-value horticultural crops. In strawberry production, a major challenge is managing the two-spotted spider mite ( *Tetranychus urticae*). Researchers have successfully trained YOLOv4 models using smartphone images captured with a macro lens to detect not only the spider mites and their eggs but also two key predatory mite species used for their control, *Phytoseiulus persimilis* and *Neoseiulus californicus* ( Zhou et al., 2023). The system achieved a mean average precision of over 93%, demonstrating its potential as a rapid, in-field tool for monitoring the crucial pest-predator balance. Other research has focused on identifying beneficial insects such as bees, ladybugs, and parasitoid wasps, recognizing their importance for both pollination and pest control ( Hassell and Waage, 1984; Rondoni et al., 2021).

The hardware for these vision systems is becoming increasingly accessible and versatile. High-resolution RGB cameras are the most common, but they are often supplemented with multispectral cameras that can detect changes in plant health reflected in near-infrared light, or thermal cameras that can detect heat signatures from insect infestations. These sensors can be deployed in stationary monitoring stations within a greenhouse or mounted on mobile platforms like drones and ground robots for broad-area coverage ( Cuthbertson and Walters, 2005).

*2.1.2 Advanced sensing technologies: acoustic and hyperspectral monitoring*

Beyond the visible spectrum, other sensing modalities are being explored to provide even earlier and more comprehensive pest detection. Acoustic monitoring involves the use of sensitive microphones and vibration sensors to detect the sounds produced by insects, particularly those that are hidden within plant tissues, wood, or soil (cryptic pests). Larvae feeding and moving within a plant stem or fruit generate subtle acoustic signatures that can be picked up by these sensors. AI algorithms, particularly machine learning classifiers, are then used to analyze these acoustic signals, filtering out background noise and identifying the unique patterns associated with specific pest species ( Kasinathan and Uyyala, 2021). This technology offers a non-destructive method for detecting infestations before external signs of damage are visible. While most research has focused on pests, the same principles can be applied to monitoring beneficial organisms. For instance, acoustic traps have been used to monitor parasitoid flies that are attracted to the calling songs of their cicada hosts, demonstrating the potential to track natural enemy activity through sound ( Farris et al., 2008).

Hyperspectral imaging (HSI) is another powerful, non-invasive technique ( Blanco et al., 2023). Unlike standard RGB cameras that capture three broad color bands, HSI sensors capture hundreds of narrow spectral bands across a wide range of the electromagnetic spectrum. This detailed spectral information can reveal subtle changes in a plant’s physiological and biochemical state. When a plant is under stress from a pest infestation, its spectral reflectance profile changes, often long before symptoms like yellowing or necrosis become visible to the naked eye or an RGB camera. Machine learning models are essential for analyzing the vast datasets generated by HSI ( Benos et al., 2021). They can be trained to recognize the specific spectral signatures associated with different types of pest damage, allowing for pre-symptomatic detection and enabling growers to intervene at the earliest possible stage of an infestation ( Scutelnic et al., 2026).

*2.1.3 Unmanned Aerial and Ground Vehicles (UAVs/UGVs) for automated scouting*

To apply these advanced sensing technologies at a commercial scale, they are increasingly being integrated with autonomous mobile platforms. Unmanned Aerial Vehicles (UAVs), or drones, are particularly well-suited for scouting large, open-field horticultural crops and orchards. A single drone equipped with a high-resolution multispectral camera can survey hundreds of acres in an hour, a task that would take a human scout days to complete. The imagery collected is geotagged, allowing AI software to stitch it together into a detailed map of the entire field. Machine learning algorithms then analyze this map to identify “hotspots” of potential pest activity or plant stress, which can be flagged for closer inspection ( Sabrina et al., 2022) This approach does not eliminate the need for human expertise but rather directs it more efficiently, allowing scouts to go directly to problem areas instead of walking random transects.

For crops with complex three-dimensional canopies, such as those in greenhouses or in open field, Unmanned Ground Vehicles (UGVs), or ground robots, offer a complementary solution ( Li et al., 2025). These robots can navigate between crop rows, capturing high-resolution, close-range imagery from below or within the plant canopy, areas that are often missed by aerial surveys. These UGVs can be equipped with a suite of sensors, including RGB cameras, hyperspectral imagers, and robotic arms for physical sampling, providing a highly detailed, plant-level assessment of pest and disease pressure.

### 2.2 Predictive analytics for proactive biocontrol

While enhanced surveillance provides a clearer picture of the present state of the agroecosystem, the true power of AI lies in its ability to use this data to predict the future ( Shepherd et al., 2020). Predictive analytics, driven by machine learning, is enabling a fundamental shift in IPM from a reactive posture - responding to problems as they appear- to a proactive and preventative one ( Vamathevan et al., 2019). This is particularly crucial for biological control, where success often depends on introducing BCAs before pest populations become well-established. This move toward prediction also represents a significant step in managing ecological complexity. Simple predictive models may correlate environmental factors with pest numbers, offering a forecast of likely population growth ( Castaño-Serna et al., 2025). However, a key challenge for biocontrol has always been its perceived unpredictability, which stems from the intricate and often non-linear dynamics of predator-prey interactions. Traditional ecological models, such as the Lotka-Volterra equations, are often too simplified to be of practical use in a variable farm environment. Advanced AI-driven models, in contrast, can handle vast, heterogeneous datasets and can learn the complex, non-linear relationships that define these biological interactions directly from field data. This allows for the development of empirically-grounded simulations that can be used for “what-if” scenario planning ( Shi et al., 2025). A grower, equipped with such a tool, could ask questions like, “Given the current pest-predator ratio and the 10-day weather forecast, what is the probable outcome if I release 1,000 predatory mites today versus 2,000 next week?” By making the outcomes of biological control more predictable and quantifiable, AI can significantly de-risk its adoption and encourage growers to rely on these sustainable strategies with greater confidence.

*2.2.1 Machine learning models for pest outbreak forecasting*

Machine learning models are being developed to forecast pest outbreaks by analyzing complex interactions between historical pest data, real-time environmental conditions, and climate predictions ( Liakos et al., 2018). Algorithms such as Decision Trees, Support Vector Machines (SVMs), and various types of Neural Networks (including Recurrent Neural Networks, or RNNs, which are well-suited for time-series data) are trained on datasets that link weather variables (like temperature, humidity, and precipitation) to past pest population dynamics. Once trained, these models can take current and forecasted weather data as input and predict the likelihood of a pest population reaching its action threshold in the near future ( May & Hassell, 1988). This provides an early warning system, giving growers a critical window of time to plan and implement preventative control measures, such as an inoculative release of BCAs, before an outbreak occurs. The integration of data from remote sensing platforms, such as satellite imagery that measures vegetation indices over large areas, allows these predictive models to operate at a landscape scale ( Rani et al., 2018; Reynolds and Riley, 2002). This can help in identifying regional pest pressure and predicting the movement of migratory pests, as demonstrated in systems for monitoring desert locusts in Africa and fall armyworm in South America.

*2.2.2 Modeling pest-natural enemy dynamics for ecological insight*

Beyond simply forecasting pest numbers, more sophisticated models are being developed to simulate the dynamic interactions between pests and their natural enemies ( Kawakita and Takahashi, 2022). These models aim to capture the complex ecological processes that govern the success or failure of a biological control program ( Mills and Getz, 1996). Using approaches like system dynamics or machine learning, these models can incorporate factors such as pest hibernation cycles, the non-linear effects of releasing BCAs at different densities, and the competitive interactions that occur when multiple pest and natural enemy species are present in the same system ( Gao et al., 2024).

For instance, a study used logistic regression models based on extensive field data from tomato greenhouses to predict the probability of achieving successful biological control based on the observed densities of pests (whiteflies, spider mites) and their respective predators. The resulting models provided clear isoclines showing the pest-predator ratios at which the pest population was likely to increase versus decrease, offering a direct decision-making tool for growers ( Tang and Cheke, 2008) These advanced models provide deep ecological insights that can inform more strategic and effective biocontrol programs, moving beyond simple releases to a more nuanced management of the agroecosystem.

### 2.3 AI-driven optimization and deployment of biocontrol agents

 The final step in the IPM process is control. AI is revolutionizing this stage by closing the loop from sensing and prediction to precise, automated action. By integrating real-time data with optimization algorithms and robotic systems, AI ensures that the right biocontrol agent is delivered to the right place, at the right time, and in the right amount. This level of precision maximizes the efficacy of BCAs while minimizing costs and labor. This integration of technologies is creating what can be conceptualized as a “digital nervous system” for the agroecosystem (  Figure 1). IPM has always been envisioned as an intelligent, responsive management system. Historically, however, the “brain” and “sensory apparatus” of this system was the human grower, who had to manually perform all the steps of data collection, interpretation, decision-making, and action. This human-centric process created inherent bottlenecks in terms of speed, scale, and the ability to process complex information. The convergence of AI and the Internet of Things (IoT) is now externalizing this nervous system.

**Figure 1.**
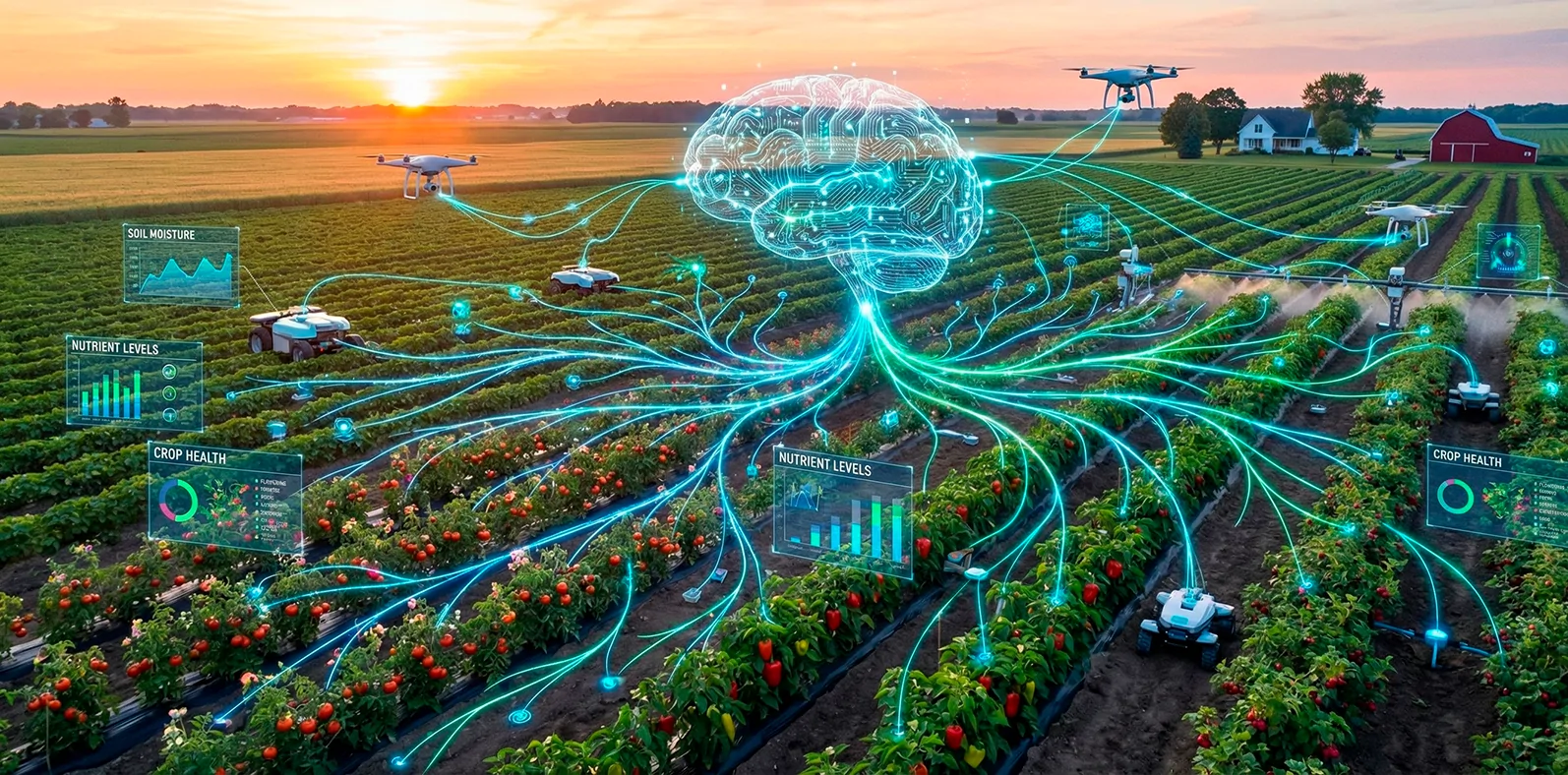
A concept of a “digital nervous system” in horticulture.

In-field sensors and cameras act as the distributed “sensory organs,” constantly gathering data ( Bu and Wang, 2019; Miller et al., 2025). This data flows to a central processing unit (either in the cloud or on an edge computing device) where AI and machine learning models serve as the “brain,” analyzing the information and making decisions. Finally, commands are sent to automated systems like drones and robotic sprayers, which function as the “motor system,” carrying out the prescribed action with high precision. This creates a fully realized, automated “sense-decide-act” loop for pest management. This is not merely an incremental improvement on existing IPM; it represents the technological actualization of the IPM concept at a scale and speed previously unimaginable, paving the way for truly autonomous and ecologically-attuned crop protection.

*2.3.1 Optimizing release strategies: timing, rates, and locations*

One of the most critical factors in the success of augmentative biological control is the release strategy. Releasing too few BCAs, too late, or in the wrong locations can result in control failure and wasted investment. AI-powered optimization algorithms can analyze the data gathered from surveillance and predictive models to calculate the ideal release plan. These systems can take into account a multitude of variables, including the current pest density and distribution (from monitoring data), the predicted rate of pest population growth (from forecasting models), the developmental rates of both the pest and the BCA at current and forecasted temperatures, and the cost of the BCAs ( Yang et al., 2026). By processing these factors, the AI can recommend a site-specific and dynamic release schedule that specifies the optimal timing for the first release, the required release rate (e.g., mites per square meter), and the ideal spatial distribution pattern to target pest hotspots effectively. This data-driven approach replaces generalized, “one-size-fits-all” recommendations with customized strategies that maximize the probability of success and provide the best return on investment. Furthermore, AI shows potential in enhancing classical biological control by aiding in the selection of the most suitable biocontrol agents from their native range and in selective breeding programs to improve key traits like environmental tolerance or host-finding ability in existing BCAs.

*2.3.2 Robotic and drone-based systems for precision application of macrobials*

The physical act of releasing macrobial BCAs, such as predatory mites or parasitic wasps, can be a significant logistical challenge, especially over large areas. Manual application is slow, labor-intensive, and often results in uneven distribution. Robotic systems are being developed to automate this process with far greater efficiency and precision. UAVs are at the forefront of this technology. Drones equipped with specialized, temperature-controlled hoppers and calibrated release mechanisms can distribute beneficial insects and mites quickly and uniformly ( Talaeizadeh et al., 2025). A single drone can cover an acre in under a minute, a task that might take a person up to an hour. This speed is crucial for timing releases to coincide with the most vulnerable stage of the pest’s life cycle. Drones can also easily access tall tree canopies in orchards or navigate difficult terrain, ensuring complete coverage where ground access is limited. For more targeted, plant-by-plant applications, UGVs are being developed. These ground robots can navigate autonomously through crop rows and use robotic arms to place BCA-containing sachets or pheromone dispensers directly onto individual plants ( Zhang et al., 2026). Agricultural drone operations in the EU are governed by a complex “dual-pillar” framework involving aviation safety and environmental protection ( Szira, 2025). From an aviation perspective, spraying drones typically operate in the ‘specific’ category under Regulation (EU) 2019/947, requiring operational authorization or adherence to Pre-Defined Risk Assessments (PDRA) due to the risks of dispensing chemicals ( Guebsi et al., 2024). However, the major bottleneck remains Directive 2009/128/EC, which bans aerial spraying unless a Member State grants a specific derogation ( Szira, 2025). While the proposed Sustainable Use Regulation (SUR) was withdrawn in 2024, recent initiatives in late 2025 aim to amend the Directive to explicitly recognize drones as precision farming tools, distinct from conventional aircraft, to facilitate their adoption.

In greenhouses, automated dispensers like the Entomatic™ system can be mounted on existing pipe-rail trolleys or other mobile platforms to release predatory mites. These systems have been shown to reduce the labor costs associated with BCA application by as much as 90% while improving the uniformity of distribution ( Potamitis and Rigakis, 2015).

*2.3.3 Precision spraying technologies for microbial and semiochemical agents*

For BCAs that are applied as sprays, such as microbial biopesticides (e.g., *Bacillus thuringiensis*, *Beauveria bassiana, Bacillus velezensis, etc*) and natural substances ( Ortiz & Sansinenea., 2022; Liu et al., 2003; Yi et al., 2022; Rabbee et al., 2023), AI-driven precision spraying technology offers a way to drastically reduce input, use while maintaining efficacy. These “smart sprayers” use real-time computer vision to differentiate between crops and weeds ( Rustia et al., 2021; Todorov & Saeys, 2019), or between healthy and infested plant tissue. Systems like “John Deere’s See & Spray” or the “Ecorobotix ARA” use cameras and AI algorithms to identify a target (e.g., a weed or a diseased leaf ) and then actuate individual spray nozzles to apply a micro-dose of product only to that specific target, leaving the surrounding crop and soil untouched ( Anne et al., 2024). This technology can reduce the total volume of pesticide or biopesticide applied by up to 90% compared to broadcast spraying ( Van Steenbergen et al., 2024). This not only saves costs but also significantly minimizes the environmental impact and helps preserve beneficial organisms in the field. This targeted approach can be further enhanced with technologies like electrostatic spraying, which imparts an electrical charge to the spray droplets, causing them to adhere more effectively to plant surfaces and reducing drift and runoff. Similarly, AI can guide the application of sprayable pheromone formulations for mating disruption, targeting areas with the highest predicted pest pressure.

### 2.4 Integrated AI-IPM Decision Support Systems (DSS)

The culmination of these AI-powered technologies is the development of integrated Decision Support Systems (DSS; Power, 2002; Shtienberg, 2013). A modern agricultural DSS acts as the central hub, ingesting data from a wide array of sources (in-field sensors, drone imagery, weather stations, predictive models, and user-inputted scouting reports) and translating this complex information into clear, actionable recommendations for the grower ( Hagelskjær &. Jørgensen, 2003; Bregaglio et al., 2022).

*2.4.1 Architecture of modern agricultural DSS*

Unlike earlier DSS, which often relied on static models, today’s systems are dynamic and adaptive ( Bregaglio et al., 2022). They are built on cloud-based platforms and leverage machine learning to continuously learn and refine their recommendations based on new data. The user interface is typically a smartphone or tablet application, making the information highly accessible ( Sprague and Carlson, 1982). These systems move beyond simple alerts (e.g., “Pest threshold exceeded”) to provide more sophisticated support, such as “what-if” scenario planning, cost-benefit analysis of different control options, and optimized weekly management schedules. By encapsulating scientific knowledge and real-time data into an easy-to-use format, these DSS empower growers to make more informed, timely, and effective pest management decisions ( Magarey et al., 2002; Shtienberg, 2013).

*2.4.2 Case studies in horticultural systems*

The practical application and benefits of integrated AI-IPM systems are best illustrated through case studies across various horticultural sectors. These examples demonstrate the technology’s effectiveness in real-world production environments, providing quantitative evidence of its impact on pest control, resource use, and economic outcomes.

In greenhouse horticulture, where environmental conditions are controlled, AI systems have shown remarkable success. AI algorithms can detect early signs of pests like whiteflies, thrips, and spider mites, as well as diseases, and alert the grower ( Kakkar et al., 2016). Critically, the company is expanding the system’s capabilities to track the populations of beneficial insects, providing a direct measure of biocontrol activity.

On the other hand, in field-grown strawberries, for instance, the development of smartphone-based deep learning tools for mite detection is a game-changer for IPM ( Kaya & Gürsoy, 2025). By enabling growers to quickly and accurately assess the ratio of pest mites to predatory mites on a leaf, these tools provide the precise data needed to decide whether a release of additional predators is necessary or if a chemical intervention can be avoided. The economic implications are substantial.

The horticultural sector is currently experiencing a fundamental paradigm shift driven by the convergence of Generative AI (GenAI) and Agentic AI, transitioning the industry from predictive analytics to autonomous reasoning. Recent literature identifies 2025 as the onset of “cognitive horticulture,” characterized by Agentic AI systems that possess the capacity to autonomously perceive environmental variables, reason about agronomic requirements, and execute interventions in real-time ( Srinivasu et al., 2026). Unlike previous static models, these agents operate in a “human-on-the-loop” capacity, managing the biological complexity of tomato greenhouses and apple orchards with minimal human oversight.

Concurrently, Large Language Models (LLMs) are revolutionizing data accessibility and synthesis. New frameworks, such as Multi-Modal LLMs, are democratizing expert knowledge by functioning as conversational extension agents for growers ( Zhu et al., 2025). Furthermore, GenAI addresses the critical challenge of data scarcity in specialty crops; the generation of synthetic datasets is now enabling the training of robust computer vision models for fruit detection and disease diagnosis without the need for extensive manual labeling. Pearson (2025) posits that this evolution from automation to agency represents the most significant technological advancement in the field’s recent history, promising unprecedented efficiency in high-value fruit and vegetable production.

## 3. Discussion

The evidence presented in the preceding section demonstrates a clear and compelling trajectory: artificial intelligence is not merely an incremental improvement for horticultural IPM but a transformative force, particularly in its capacity to elevate biological control from a niche strategy to a mainstream, reliable, and data-driven practice. The synergy between AI’s analytical power and biocontrol’s ecological principles offers a potent solution to the complex challenges facing modern horticulture ( Salybekova et al., 2024; Chaudhary et al., 2025). However, the path from technological potential to widespread, equitable adoption is fraught with significant hurdles. This section synthesizes these findings, discussing how AI addresses key IPM challenges, the profound implications of this technological convergence, the barriers that must be overcome, and the critical research gaps that need to be addressed to realize a future of intelligent, sustainable pest management.

### 3.1 Synthesizing the synergy: how AI addresses key IPM challenges

The true value of AI in IPM becomes evident when its capabilities are mapped directly onto the pressing challenges of climate change, pesticide resistance, and invasive species ( Venette and Hutchison, 2021). AI provides the adaptive and predictive tools necessary to manage the increased complexity and uncertainty that these challenges introduce ( Raj & Prahadeeswaran, 2025; Omotayo et al.,2025) In the face of climate change, which makes pest dynamics more volatile and unpredictable, AI’s predictive modeling capabilities are invaluable ( Sharma, 2021; Welch & Harwood, 2014). By integrating real-time meteorological data from on-farm sensors and regional weather forecasts with historical pest population data, machine learning models can generate short-term outbreak predictions that are tailored to local conditions ( Karuppaiah & Sujayanad, 2012). This allows growers to adapt their management strategies dynamically, moving away from rigid, calendar-based schedules that are increasingly unreliable in a changing climate ( Hatfield et al., 2011). For biological control, this means being able to time the release of natural enemies with greater precision to match the climate-altered life cycles of their target pests, ensuring the synchrony that is critical for success ( Knell, 1998; Mahr et al., 2008). AI offers a direct pathway to mitigating pesticide resistance by fundamentally reducing the selection pressure that drives it. The most effective way to slow the evolution of resistance is to decrease the frequency and intensity of chemical applications ( Eli-Chukwu, 2019). AI facilitates this by making non-chemical alternatives, especially biological control, more effective and reliable. AI-guided precision sprayers that can reduce biopesticide or conventional pesticide use by up to 90% drastically lower the exposure of pest populations to these agents ( Faria and Wraight, 2007). More importantly, by providing the tools to accurately monitor pest-predator ratios and optimize BCA releases, AI empowers growers to rely on biocontrol as a primary strategy, reserving chemicals as a true last resort. This multifaceted reduction in chemical dependence is the most sustainable long-term resistance management strategy.

Against the threat of invasive species, AI-powered surveillance acts as a critical early warning system. The rapid and accurate identification of a newly introduced pest is essential for enabling a swift containment or eradication response before the population becomes established. Networks of smart traps, powered by computer vision and connected via the IoT, can create a large-scale, automated monitoring grid that can detect an invasive species far faster than traditional manual trapping networks ( Ramu et al., 2025). Furthermore, once an invasive pest is detected, AI can help model its potential spread based on climate and landscape data, guiding survey and control efforts to the highest-risk areas.

### 3.2 From data to action: the transformative potential of AI-enhanced biocontrol

The integration of AI technologies (sensors, computer vision, machine learning, and robotics) is creating a closed-loop system for pest management that mirrors a biological nervous system ( Reynolds and Riley, 2002). This is perhaps the most profound implication of the current technological trend. If the agroecosystem is the “body,” then the network of sensors and cameras acts as its “sensory organs,” continuously gathering data on its internal state. The AI and machine learning models, whether running on cloud servers or on-site edge computing devices, function as the “brain,” processing this sensory input, identifying patterns, and making complex decisions. Finally, the autonomous drones and ground robots act as the “motor system,” executing the brain’s commands by precisely delivering the required intervention (be it a predatory mite or a micro-dose of a biopesticide) to the exact location where it is needed ( Guebsi et al., 2024). This creation of a digital “sense-decide-act” loop represents the technological actualization of the IPM philosophy. IPM has always been conceptualized as an intelligent, knowledge-based process, but its implementation has been constrained by the limitations of its human operators in terms of speed, scale, and cognitive capacity to process complex, interacting variables ( Shepherd et al., 2020). AI removes these constraints, allowing IPM principles to be applied with a speed and precision that were previously unimaginable. This moves pest management beyond human-guided precision and towards true autonomous control, where the system can self-regulate based on real-time ecological feedback.

### 3.3 Overcoming the hurdles: barriers to adoption and implementation

Despite the immense potential, the transition to AI-driven biocontrol is not without significant challenges. These barriers are not only technological but also deeply rooted in the socio-economic and ethical fabric of agriculture ( Sparrow et al., 2021).

*3.3.1 Technological and data-related challenges*

The performance of any AI system is fundamentally dependent on the quality and quantity of the data used to train it ( Sykuta, 2016) This presents a major bottleneck for agricultural applications. High-quality, accurately labeled datasets of pests and, even more so, beneficial insects are scarce and expensive to create ( Berisha et al., 2026) A model trained to identify spider mites in a Dutch greenhouse may perform poorly when deployed in a Spanish strawberry field due to differences in lighting, crop variety, and background clutter ( Sunderland, 1988). This problem of model generalization is a critical hurdle that requires the development of more robust algorithms and the creation of larger, more diverse, and standardized public datasets. Furthermore, the deployment of these systems requires significant hardware and infrastructure. High-speed internet connectivity is not universally available in rural areas, which can limit the use of cloud-based AI models. While edge computing (processing data directly on-device) offers a solution, it requires specialized, powerful hardware. The cost of high-resolution cameras, advanced sensors, and robotic platforms remains a substantial barrier to entry for many growers.

*3.3.2 Socio-economic and ethical implications*

The economic and social impacts of AI in agriculture are complex and warrant careful consideration. The high initial investment cost of AI technologies risks creating a “digital divide,” where large, well-capitalized agribusinesses can leverage these tools to increase their efficiency and market share, while small and medium-sized family farms are left behind ( Trendov et al., 2019). This could accelerate the trend of farm consolidation and reduce the diversity of the agricultural landscape. The automation of tasks previously performed by human scouts and laborers raises legitimate concerns about labor displacement ( Oliveira et al., 2021). While AI may create new, higher-skilled jobs in areas like data science and robotics, there is a risk of significant disruption for the existing agricultural workforce. A just transition will require substantial investment in education and retraining programs to equip rural communities with the skills needed for the farms of the future. Finally, a host of ethical questions must be addressed to ensure that AI is deployed responsibly ( Ryan, 2023). Data ownership and privacy are paramount: who owns the vast amounts of data generated on a farm, and how is it used? If data is controlled by a few large tech companies, farmers could become dependent on proprietary systems, losing autonomy over their own operations ( McCown , 2002).

Algorithmic transparency is another key issue. Many deep learning models operate as “black boxes,” making it difficult to understand how they arrive at a particular recommendation ( Sit et al., 2020). This lack of explainability can erode farmer trust and make it difficult to diagnose and correct errors. As some cases have shown, blindly following an AI recommendation that is not attuned to local regulations or specific agronomic conditions can lead to illegal pesticide applications or severe crop damage, highlighting the continued need for human oversight and expertise.

### 3.4 Research gaps and future directions for an AI-Integrated biocontrol future

To navigate these challenges and fully unlock the potential of AI-enhanced biocontrol, future research and development should focus on several key areas:

• Broadening the focus to beneficials **:** a disproportionate amount of research has focused on pest detection ( Dong et al., 2022). A concerted effort is needed to create large, public datasets and train robust models for a much wider range of beneficial insects and mites, including their different life stages. This is essential for developing true ecosystem-based monitoring systems.
• Optimizing conservation and classical biocontrol: current AI applications are heavily skewed towards improving augmentative biocontrol (the periodic release of commercially reared agents). There is a significant research gap and opportunity in applying AI to enhance other biocontrol strategies. For example, AI could be used to design and manage on-farm habitats (e.g., flowering strips and use of banker plants) to support native natural enemies (conservation biocontrol) or to model and predict the establishment success of an imported agent in a new environment (classical biocontrol; Hajek, 2004; Szitar et al., 2022).
• Understanding microbiome interactions: the introduction of microbial BCAs can have complex and sometimes unpredictable effects on the native soil and plant microbiomes. AI and machine learning are powerful tools for analyzing the complex metagenomic data needed to understand these interactions, ensuring that microbial releases do not have unintended negative ecological consequences ( Ortiz and Sansinenea, 2022).
• Developing “Explainable AI” (XAI): to build trust and facilitate adoption, the “black box” nature of many AI models must be addressed. Research into Explainable AI (XAI) aims to create systems that can articulate the reasoning behind their predictions and recommendations.
• Multi-modal data fusion: the most robust and accurate systems of the future will likely integrate data from multiple sensing modalities. Fusing information from visual cameras, hyperspectral sensors, acoustic monitors, and environmental data streams will provide a more complete and resilient picture of the agroecosystem, leading to better-informed management decisions.

## 4. Conclusions

 The global horticultural industry stands at a critical juncture, compelled to meet the demands of a growing population while simultaneously navigating the urgent need for environmental sustainability. This review has examined how the integration of artificial intelligence with biological control agents is forging a new and promising path forward, offering a powerful alternative to the unsustainable model of chemical-dependent agriculture. The synthesized evidence from a vast body of scientific literature clearly indicates that this convergence is not merely an incremental improvement but a fundamental transformation of Integrated Pest Management. By leveraging the analytical power of AI and the precision of robotics, this next-generation “IPM 5.0” is closing the long-standing gap between the principles and the practice of IPM, creating systems that are more predictive, precise, efficient, and ecologically sound ( Araújo et al., 2021; Wu et al., 2025).

We have demonstrated how AI technologies are revolutionizing each tier of the IPM framework. They are replacing laborious manual scouting with automated, real-time surveillance that can identify pests, diseases, and assess crop health with superhuman accuracy. They are enhancing pest stewardship by ensuring the right pest is identified, the right control method is selected, and the right action is taken at the right time. This is accomplished through AI-driven decision support systems that synthesize complex variables and robotic platforms that execute interventions with surgical precision. This synergy creates powerful, positive ecological feedback loops; by minimizing the use of broad-spectrum pesticides, these systems conserve and enhance populations of beneficial organisms, such as pollinators and natural enemies, which in turn contribute to improved crop yields and greater agroecosystem resilience ( Bishop et al., 2023). The future promises even more profound advancements, with swarm robotics enabling decentralized, collaborative pest control and the fusion of AI and genomics allowing for the rational design of next-generation biological control agents with enhanced efficacy and environmental safety.

However, the path to widespread and equitable adoption is not without significant obstacles. High initial capital costs, a persistent “scalability gap” between controlled environments and open fields, a critical lack of high-quality and diverse training data (especially for beneficial species) and the need for new skills and infrastructure all present formidable challenges. Overcoming these hurdles will require a concerted and collaborative effort from all stakeholders, from researchers and technology developers to policymakers, growers and technical advisors too. Furthermore, the profound ethical and social implications surrounding data ownership, labor displacement, algorithmic bias, and biosecurity must be addressed proactively through thoughtful governance and inclusive design processes ( Ryan et al., 2023).

The journey towards a fully realized vision of AI-integrated IPM will be complex, requiring sustained investment, interdisciplinary innovation, and robust collaboration. Yet, the potential rewards, a horticultural sector that is more productive, profitable, and in harmony with the environment, are immense. By embracing these advanced strategies and thoughtfully navigating their complexities, we can cultivate a future where technology and ecology work in concert to sustainably and securely feed a growing world.

Note: Generative AI (Google Gemini ver. 3.1 Pro) was used, in accordance to EU AI Act in force, to generate  Figure 1, as the “digital nervous system” in horticulture should be considered a representation of a comprehensive evolution of the knowledge and technology networks necessary in modern, resilience and sustainable agroecosystems.

## Ethics and consent

Ethical approval and consent were not required.

## Data Availability

No data are associated with this article.
