# Supplementary material for: New challenges in horticultural IPM: artificial intelligence can enhance the use of biocontrol agents
Source: Open Res Eur. 2026 Jun 15;6:204. [Version 1] doi: 10.12688/openreseurope.23884.1 (PMC13679776; doi:10.12688/openreseurope.23884.1)
Supplement: Peer review history

# Reviewer report: Reviewer response for version 1

**DOI:** 10.21956/openreseurope.25859.r77352

**Published:** 2026-08-06

**Authors:** Rashmi Mishra

**Affiliations:** 1 University of Technology and Applied Sciences, Ibra, Oman

The manuscript focuses on a timely and relevant topic; however, several aspects require improvement to develop its systematic value and clarity. The author is advised to arrange the keywords under the abstract in alphabetical order, following standard journal formatting requirements. The author may consider revising the objective to explicitly state that the review aims to examine how AI can strengthen IPM through improved precision, real-time monitoring, predictive decision-making, and ecological sustainability. The description of the four-tiered IPM framework would benefit from the insertion of a diagram or flowchart. This would significantly improve readers' understanding of the interconnections among the different stages and the integration of AI technologies within the IPM framework. The manuscript would be strengthened by incorporating more recent and updated references on AI-assisted pest detection, machine learning, computer vision, remote sensing, IoT-enabled monitoring systems, and decision-support tools in IPM. The research gaps are presently listed as bullet points without adequate justification from the literature. Each gap should be critically discussed and supported by appropriate, recent in-text citations. The researcher is encouraged to synthesize the existing literature to clearly demonstrate how these gaps emerge from previous studies and why they deserve further investigation.

Is the review written in accessible language?

Yes

Are all factual statements correct and adequately supported by citations?

Partly

Are the conclusions drawn appropriate in the context of the current research literature?

Partly

Is the topic of the review discussed comprehensively in the context of the current literature?

Yes

Reviewer Expertise:

Management and Business, Quality Assurance, Economics, Marketing, HRM

I confirm that I have read this submission and believe that I have an appropriate level of expertise to confirm that it is of an acceptable scientific standard, however I have significant reservations, as outlined above.

# Reviewer report: Reviewer response for version 1

**DOI:** 10.21956/openreseurope.25859.r77942

**Published:** 2026-07-30

**Authors:** Dr. Mushtaque Ahmed Rahu

**Affiliations:** 1 Electronic Engineering, Quaid-e-Awam University of Engineering, Science and Technology, Nawab Shah, Nawabshah, Pakistan

**Brief Summary:**

 The titled review paper "New challenges in horticultural IPM: artificial intelligence can enhance the use of biocontrol agents" in touch of AI is good, It includes biocontrol basics, incorporation, trends and challenges, a literature survey.

 Paper is sound, having good knowledge for readers of the said domain, applications of AI, agriculture.

 I strongly recommend this paper for indexing with minor suggestions.

**Comments:**

 1. In title it would be better to change or use another suitable word instead of " new challenges".

 2. In Introduction section 1.1 too old citations are given try to improve or add latest citations please.

 3. Figure 1 is not clear try to improve and revise as per paper title and requirement.

 4. Section 2.4.2 second paragraph is not clear and proper. it must be set as per criteria complete the details in paragraph.

 5. “IPM 5.0” is not properly defined. Give briefly Are IPM 1.0/2.0/3.0/4.0 available? Please give difference mentioning in tabular form it would be better.

 6. Some citations are missing in introduction section please insert where necessary.

 7. Overall paper is good but need to polish your paper like set your introductions section specially too old citations, format of citations and references and citations missing where necessary.

Is the review written in accessible language?

Yes

Are all factual statements correct and adequately supported by citations?

Yes

Are the conclusions drawn appropriate in the context of the current research literature?

Yes

Is the topic of the review discussed comprehensively in the context of the current literature?

Yes

Reviewer Expertise:

WSN, IoT, and machine Learning applications in Smart Agriculture and water quality monitoring.

I confirm that I have read this submission and believe that I have an appropriate level of expertise to confirm that it is of an acceptable scientific standard.

# Reviewer report: Reviewer response for version 1

**DOI:** 10.21956/openreseurope.25859.r77356

**Published:** 2026-07-27

**Authors:** Yufeng Yang

**Affiliations:** 1 Department of Bioproducts & Biosystems Engineering, University of Minnesota, Twin Cities, Minnesota, USA

**Summary**

 This review paper narrates a viewpoint that artificial intelligence can address long-standing barriers to wider adoption of biological control agents (BCAs) through the framework of Integrated Pest Management (IPM). It covers biocontrol fundamentals, implementation challenges, a literature synthesis mapping AI (computer vision, machine learning, robotics, decision support systems) onto the IPM cycle, adoption barriers, and a concluding "IPM 5.0". The article is relevant to ORE's readership and merits indexing.

 The manuscript is clearly written and logically structured. It connects biocontrol ecology, agricultural AI and robotics, and regulatory policy with discussions on their synergic or intervening relationships. The identified research gaps (models for beneficial organisms, conservation/classical biocontrol, explainable AI, multi-modal data fusion) are a genuine value-add. My recommendation is Approved with Reservations, concerning weakness in Figure 1, dated sourcing in section 1.3, and a few editorial issues.

**Comments**

 1. Figure 1 needs revision. The “digital nervous system” figure is visually appealing but could use some improvement. Text labels are redundant (e.g., "crop health" and "nutrient level" appear to duplicate), while some components the manuscript explicitly discusses are missing from the figure: weather data as inputs and the AI model/system. Given that the paper argues that AI enables fast prediction and response, readers might benefit from this figure or another separate figure depicting the prediction pipeline: what input data is, where and how it is processed (edge vs. cloud), what types of algorithm are used, and how outputs reach the end device. 

 2. Section 1.3 relies heavily on dated citations. The subsection on climate change, resistance, and invasive species describes emerging and current problems, but most supported citations that are more than a decade old. A few recent sources would help readers judge whether the trends described are still accurate and how they may have shifted.

 3. Table 1 lists BCA mechanisms but not much else. Would it be useful to also note the pros/cons of each category? "Pores germinate" should read "spores germinate."

 4. In Section 2.4.2, “the company” was mentioned with no name, which probably was an edited-out issue. Please fill in the name. 

 5.  “IPM 5.0” is introduced without grounding against previous frameworks. Are there IPM 1.0/2.0/3.0/4.0? Please clarify how IPM 5.0 differ from the previous frameworks.

 6. This Quantitative claim in the Introduction lacks a citation: “IPM can decrease pesticide applications by 30–50%.”

 7. The adopted citation formats were not consistent across the text. For example two styles were used, “(May & Hassell, 1988)” and “Reynolds and Riley, 2002)”. Please ensure that citations follow the journal's formatting requirements.

Is the review written in accessible language?

Yes

Are all factual statements correct and adequately supported by citations?

Yes

Are the conclusions drawn appropriate in the context of the current research literature?

Yes

Is the topic of the review discussed comprehensively in the context of the current literature?

Yes

Reviewer Expertise:

agricultural model simulations, AI applications in agriculture, process-based modeling, soil-plant-atmosphere interactions.

I confirm that I have read this submission and believe that I have an appropriate level of expertise to confirm that it is of an acceptable scientific standard, however I have significant reservations, as outlined above.
